# Enhanced NRF2 expression mitigates the decline in neural stem cell function during aging

**DOI:** 10.1111/acel.13385

**Published:** 2021-06-15

**Authors:** Annadurai Anandhan, Konner R. Kirwan, Mandi J. Corenblum, Lalitha Madhavan

**Affiliations:** ^1^ Department of Neurology University of Arizona Tucson AZ USA; ^2^ Pharmacology and Toxicology University of Arizona Tucson AZ USA; ^3^ Neuroscience and Cognitive Science Undergraduate Program Tucson AZ USA; ^4^ Bio5 Institute University of Arizona Tucson AZ USA; ^5^ Evelyn F McKnight Brain Institute University of Arizona Tucson AZ USA

**Keywords:** aging, neural stem cells, NRF2, redox, subventricular zone

## Abstract

Although it is known that aging affects neural stem progenitor cell (NSPC) biology in fundamental ways, the underlying dynamics of this process are not fully understood. Our previous work identified a specific critical period (CP) of decline in NSPC activity and function during middle age (13–15 months), and revealed the reduced expression of the redox‐sensitive transcription factor, NRF2, as a key mediator of this process. Here, we investigated whether augmenting NRF2 expression could potentially mitigate the NSPC decline across the identified CP. NRF2 expression in subventricular zone (SVZ) NSPCs was upregulated via GFP tagged recombinant adeno‐associated viral vectors (AAV‐NRF2‐eGFP), and its cellular and behavioral effects compared to animals that received control vectors (AAV‐eGFP). The vectors were administered into the SVZs of aging rats, at time points either before or after the CP. Results indicate that animals that had received AAV‐NRF2‐eGFP, prior to the CP (11 months of age), exhibited substantially improved behavioral function (fine olfactory discrimination and motor tasks) in comparison to those receiving control viruses. Further analysis revealed that NSPC proliferation, self‐renewal, neurogenesis, and migration to the olfactory bulb had significantly increased upon NRF2 upregulation. On the other hand, increasing NRF2 after the CP (at 20 months of age) produced no notable changes in NSPC activity at either cellular or behavioral levels. These results, for the first time, indicate NRF2 pathway modulation as a means to support NSPC function with age and highlight a critical time‐dependency for activating NRF2 to enhance NSPC function.

## Graphical abstract

The current work reveals that amplifying intrinsic levels of the redox transcription factor, NRF2, before a certain critical period (CP) of aging (13–15 months), can rescue the age‐related decline in neural stem cell function in rats. This finding has fundamental implications for not only understanding neural stem cell biology, but also advantageously modulating neural stem cell function with age.

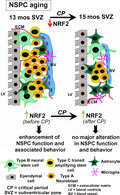

## 1 INTRODUCTION

Adult neural stem progenitor cells (NSPCs) are characterized by the ability to self‐renew and differentiate into neuronal and glial cell types in the mature nervous system (Alvarez‐Buylla & Lim, 2004; Bond et al., 2015). This cell‐level plasticity is not fixed, but rather a dynamic and highly modulated process. NSPC activity can be influenced by a range of factors, such as physical exercise, environmental enrichment, stress, and nutrition, but also importantly aging (Katsimpardi & Lledo, 2018; Mirescu & Gould, 2006; Nilsson et al., 1999; van Praag et al., 1999). In fact, aging contracts NSPC niches in the brain and significantly alters their function (Conover & Shook, 2011; Encinas et al., 2011; Liu & Rando, 2011). Given the pivotal role of stem cells in tissues with lifelong regenerative capacity such as the brain, understanding stem cell aging will be important if we are to understand aging at the organ level. More broadly, comprehending stem cell aging will also support the development of interventions that could improve both health and lifespan.

In this context, our previous studies, conducted in naturally aging rodents, identified a specific temporal pattern of change in NSPC dynamics during aging. In particular, the studies highlighted a critical time during middle age (13–15 months), when the regenerative function of NSPCs showed a striking decline (Corenblum et al., 2016; Ray et al., 2018; Schmidlin et al., 2019). The studies also determined the reduced expression of nuclear factor (erythroid‐derived 2) like 2 (or NRF2), as a key mechanism mediating this phenomenon. As such, this work provided first evidence of an important regulatory role for NRF2 in NSPC aging.

NRF2 is a redox‐sensitive transcription factor known to be essential to the cell's homeostatic mechanism (Bryan et al., 2013; Itoh et al., 2010; Suzuki & Yamamoto, 2017). NRF2 is ubiquitously expressed in most eukaryotic cells and functions to induce a broad range of cellular defenses against exogenous and endogenous stresses, including oxidants, xenobiotics, inflammatory agents, and excessive nutrient/metabolite supply. In particular, NRF2 can up‐regulate a range of classical ARE (antioxidant response element)‐driven genes, encoding major antioxidants and other detoxification enzymes. In addition to its classical function in regulating the stress response, NRF2 has been linked to cell growth, proliferation, mitochondrial and trophic functions, protein quality control, and increased lifespan (Holmstrom et al., 2013; Malhotra et al., 2010; Sykiotis & Bohmann, 2008; Tullet et al., 2008; Wakabayashi et al., 2010; Wiesner et al., 2013; Zhu et al., 2013). Our recent work adds a unique and important new face to NRF2 actions in the cell—namely the age‐relevant regulation of NSPCs (Corenblum et al., 2016; Madhavan, 2015; Ray et al., 2018; Schmidlin et al., 2019).

Given that NRF2 loss accentuates NSPC aging, in this study, we investigated whether increasing NRF2 levels could boost NSPC function with age. In particular, we studied whether inducing high intrinsic NRF2 expression can potentially mitigate the decline in NSPC regeneration during the critical middle‐age period between 13 and 15 months (mos), identified in our previous work. NRF2 was delivered to rat subventricular zone (SVZ) NSPCs through recombinant adeno‐associated viral (AAV) vectors injected either before (at 11 mos of age) or well after the critical aging period (at 20 mos of age). We find that the administration of AAV‐NRF2‐eGFP vectors before the initiation of the critical period (CP), substantially improved SVZ NSPC regeneration and associated behavioral function, as compared to controls (AAV‐eGFP delivery). On the other hand, application of AAV‐NRF2‐eGFP after the conclusion of the CP failed to significantly promote NSPC activity and function. These data establish a major governing role for NRF2 in NSPCs and support targeting the NRF2 pathway as a potential approach to advantageously modulate NSPC function with age.

## 2 RESULTS

### 2.1 Viral expression of NRF2 in aging SVZ NSPCs improves behavioral function during the critical period

**FIGURE 1:**
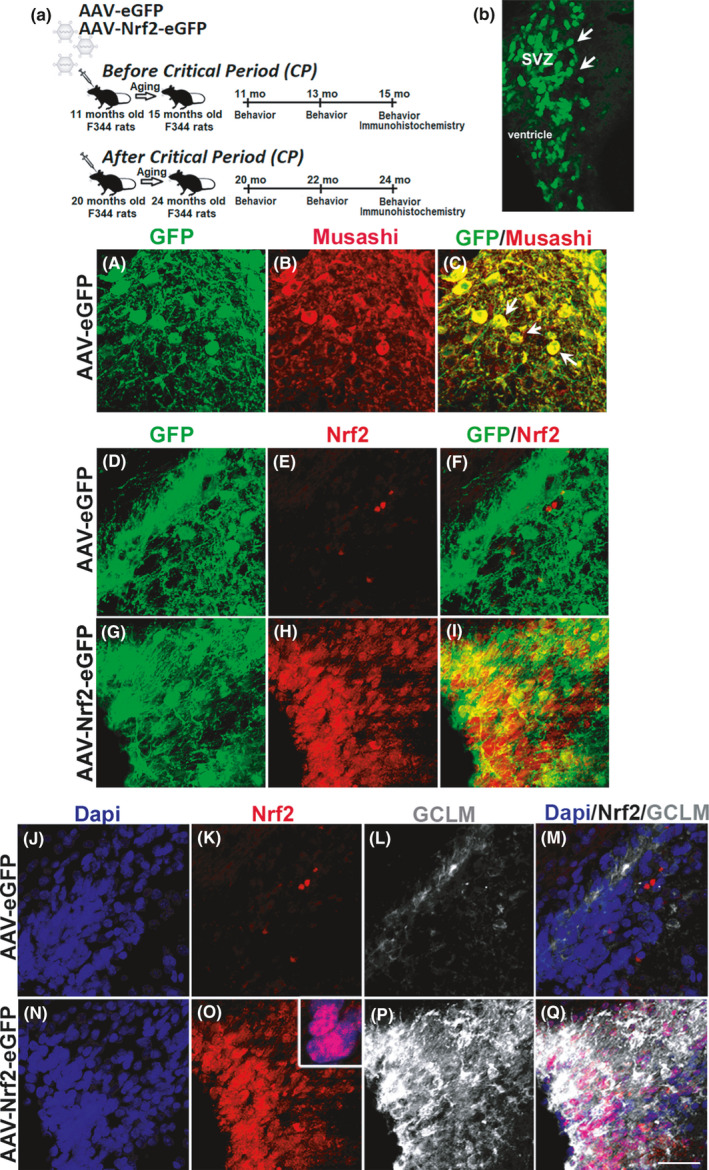
NRF2 expression and activation in the SVZ NSPCs. (a) Depicts the experimental design and timeline. (b) Shows GFP expression in SVZ cells 1 mos after AAV‐eGFP injection (white arrows). A–C Depicts confocal images of Musashi^+^ NSPCs showing high GFP expression after AAV‐eGFP transduction (arrows indicate example positive cells). GFP expressing NSPCs in the dorsolateral SVZ showed increased NRF2 expression in AAV‐Nr2‐eGFP‐injected rats (G–I, arrows) compared to rats that had received AAV‐eGFP (D–F). The NRF2 target gene, GCLM, was highly expressed in the same NRF2 overexpressing cells of AAV‐NRF2‐eGFP rats (N–Q) compared to control rats (J–M). Inset in O shows higher magnification view of NRF2/GCLM co‐labeling. Scale bar of 20 μM, applicable to images in A–Q, is drawn in Q

In order to address whether augmenting NRF2 expression can promote NSPC function during aging, recombinant adeno‐associated viral vectors tagged with a GFP reporter carrying either NRF2 (AAV‐NRF2‐eGFP), or eGFP alone (AAV‐eGFP) as a mock control, were stereotactically delivered into the SVZs of aging rats. To specifically determine the effects of rescuing NRF2 expression in the context of the critical middle‐age period (13–15 mos) identified in our previous studies, the vectors were injected into the SVZ either before (11 mos of age) or well after (20 mos of age) the CP. Subsequently, behavioral function (at 2 and 4 mos post‐viral injection) and cellular changes (4 mos post‐injection) were assessed (Figure 1a).

First, we confirmed the efficiency of viral transduction. It was found that AAV2/1 administration into two sites along the rostrocaudal extent of the lateral SVZ robustly and specifically transduces NSPCs (stereotaxic locations shown in Figure S1A,C and described in the Methods section). Strong GFP expression was noted in the rat SVZ by immunofluorescence microscopy (Figure 1b, broader views of the transduced areas are in Figure S1B,D). This high GFP expression was seen as early as 2 weeks post‐injection, with peak viral transduction reached at 1.5 mos. As shown, co‐labeling with antibodies targeting the NSPC specific antigen Musashi1 (expressed by a large population of SVZ stem and progenitor cells) indicated that AAV2/1 proficiently infected SVZ NSPCs (confocal micrographs in Figure 1A–C). Moreover, significantly increased NRF2 expression was seen in SVZ cells of animals that received AAV‐NRF2‐eGFP, as compared to GFP controls (Figure 1D–I). To ensure that NRF2 overexpression further activates downstream target genes, levels of the well‐established NRF2 target gene, glutamate–cysteine ligase modifier subunit (GCLM), in the SVZ were also assessed. As shown, GCLM expression was increased in the same SVZ cells that showed high NRF2 expression thus confirming NRF2 pathway activation (Figure 1J–Q). This level of NRF2 expression and activation appeared comparable to what was observed in 9‐ to 11‐month‐old animals as characterized previously (Corenblum et al., 2016).

**FIGURE 2:**
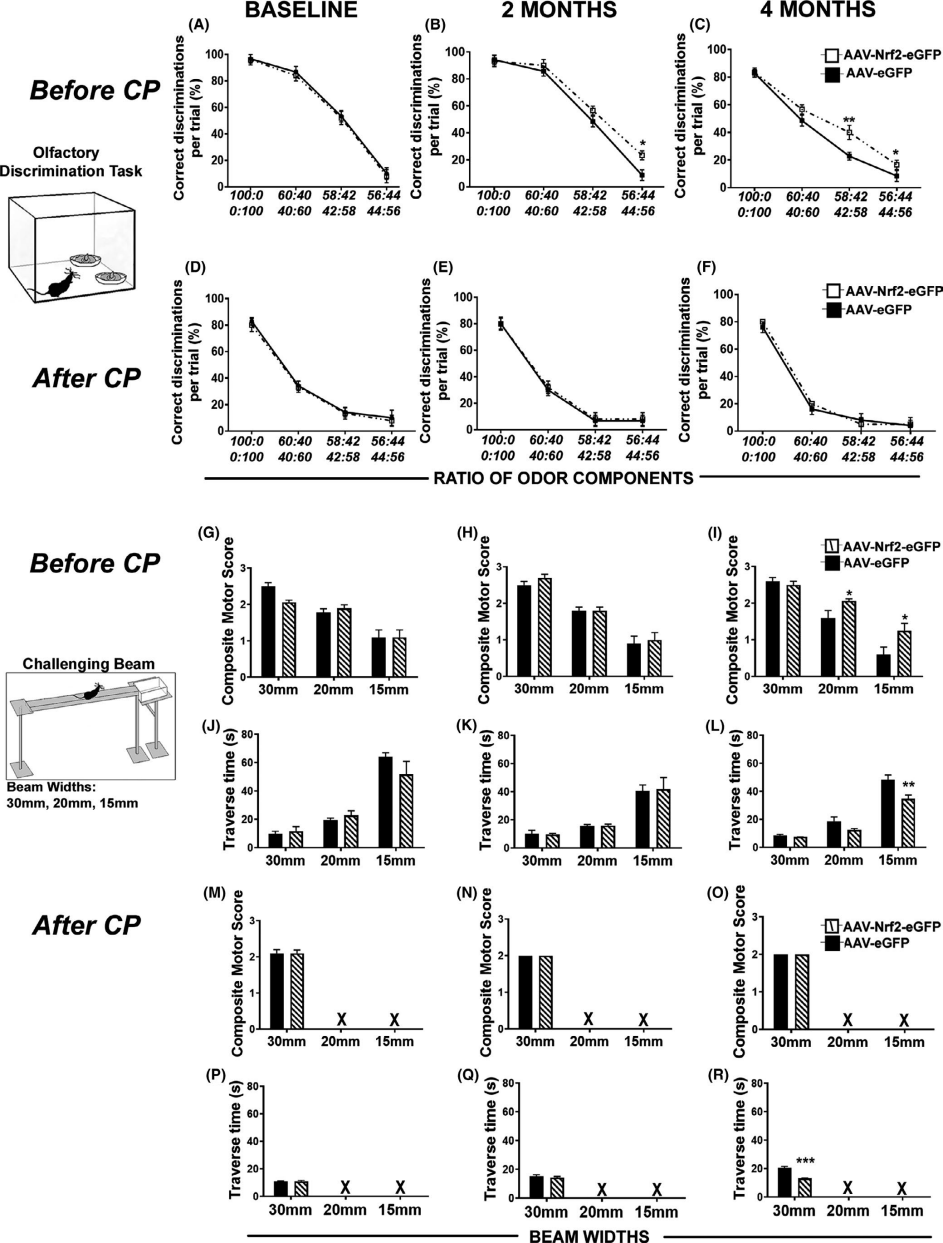
Heightened olfactory discrimination and motor abilities in NRF2 overexpressing rats. Results from baseline testing of fine olfactory discrimination (upper schematic on the left) on naïve 11‐month and 20‐month rats (aging stages before and after the CP are in (A) and (D). 11‐month‐old rats showed significantly improved abilities to discriminate between similar concentrations of odorants, 2 mos (B) and 4 mos (C) after AAV‐NRF2‐eGFP administration, compared to controls. Analysis of rats which received AAV‐NRF2‐eGFP at 20 mos of age (after the CP) showed no positive effect on fine olfactory discrimination capacities (E,F). “Ratio of odor components” label below (D–F) graphs applies to all six olfactory graphs above. [*p < 0.05, **p < 0.01, Two‐way repeated measures ANOVA with Tukey's post hoc test]. Lower schematic on the left shows the challenging beam apparatus. Younger 11‐month‐old rats showed similar composite motor scores at baseline (G) and 2 mos (H) after AAV administration. However, rats receiving AAV‐NRF2‐eGFP displayed significantly higher composite scores at 4‐month post‐viral injection when traversing the 20 mm and 15 mm beams (I). (J–L) Shows beam traversal times for AAV‐NRF2‐eGFP rats and AAV‐eGFP rats. AAV‐NRF2‐eGFP administration in older 20‐month‐old rats (after CP completion) had no effect on their composite motor scores when traversing the 30 mm beam at baseline (M), 2‐month (N) or 4‐month post‐viral injection (O). Animals were unable to cross the 20 mm or 15 mm width beams (P,Q) at this age (indicated by “x”). Beam traversal time for the 20‐month‐old AAV‐NRF2‐eGFP‐injected rats across the 30 mm beam is shown in (R). “Beam widths” label below (P–R) graphs applies to all nine beam graphs above. [*p < 0.05, **p < 0.01, ***p < 0.001, unpaired *t* test with Welch's correction]

Next, the behavioral consequences of increased NRF2 expression were analyzed. The fine olfactory discrimination task is a known measure of SVZ NSPC function that tests the animal's ability to discriminate between different ratios of [+]/good tasting coconut (COC) and [−]/bad tasting mixture of almond and denatonium benzoate (ALM) (Corenblum et al., 2016; Enwere et al., 2004; Schmidlin et al., 2019). As expected, the baseline olfactory function (i.e., prior to AAV injection) of the older 20 mos rats was significantly worse (reflected by lower scores on the Y‐axis) than the 11‐month‐old animals (Figure 2A,D). Intriguingly, as compared to the AAV‐eGFP control‐injected rats, the 11‐month‐old animals that received AAV‐NRF2‐GFP exhibited an increased capacity to discriminate between very similar ratios of COC and ALM (56:44) starting at 2 mos after injection [Figure 2B; *p *= 0.011, F_3,33_ = 217.124 (concentration), two‐way RM‐ANOVA], which became even more significant (56:44 and 58:42) by the 4 mos post‐injection time point [Figure 2c; 58:42 – *p *= 0.004, 56:466 – *p *= 0.046, F_3,33_ = 167.448 (concentration), two‐way RM‐ANOVA]. However, this effect was not seen in the 20‐month‐old animals (Figure 2D–F), supporting the importance of NRF2 activation during the CP.

We also assessed motor function via a challenging beam task to investigate potential striatal effects of increased SVZ NRF2 expression. We generated a composite score that represents the ability of an animal to cross an increasingly narrow in size set of beams without foot slip errors, scooting across, or failing to cross the beam (Figure 2G–R). While there was no significant difference in the composite scores between animals at 2 mos post‐AAV injection, the 11‐month‐old animals injected with AAV‐NRF2‐eGFP were able to successfully traverse both the 20 mm and 15 mm beams more often than their AAV‐eGFP‐injected counterparts at the 4 mos post‐injection time point (Figure 2I; 20 mm beam – *p* = 0.0395, unpaired *t* test, *t* = 2.20, df = 20; 15 mm beam – *p* = 0.028, unpaired *t* test, *t* = 2.29, df = 31). Interestingly, AAV‐NRF2‐eGFP‐injected rats also traversed the 15 mm beam in a shorter time (that is faster) than the AAV‐eGFP‐injected animals (Figure 2L; *p* = 0.007, unpaired *t* test, *t* = 3.07, df = 15) at 4 mos. No significant differences in composite scores were observed between the groups in the 20‐month‐old animals, which were only able to cross the 30 mm beam (Figure 2M–O). However, the AAV‐NRF2‐eGFP‐injected animals were found to traverse the 30 mm beam significantly more quickly at 4 mos post‐AAV injection (Figure 2R). HBSS‐injected control animals showed similar olfactory and motor abilities as GFP controls. Overall, these results indicate that activation of NRF2 in SVZ NSPCs during the critical period improves both olfactory and striatum‐based motor functions, whereas these behaviors are not rescued when NRF2 activation is delayed to an older age after the completion of the critical period.

### 2.2 SVZ NSPC proliferation and neurogenesis are enhanced following NRF2 overexpression during the critical period

**FIGURE 3:**
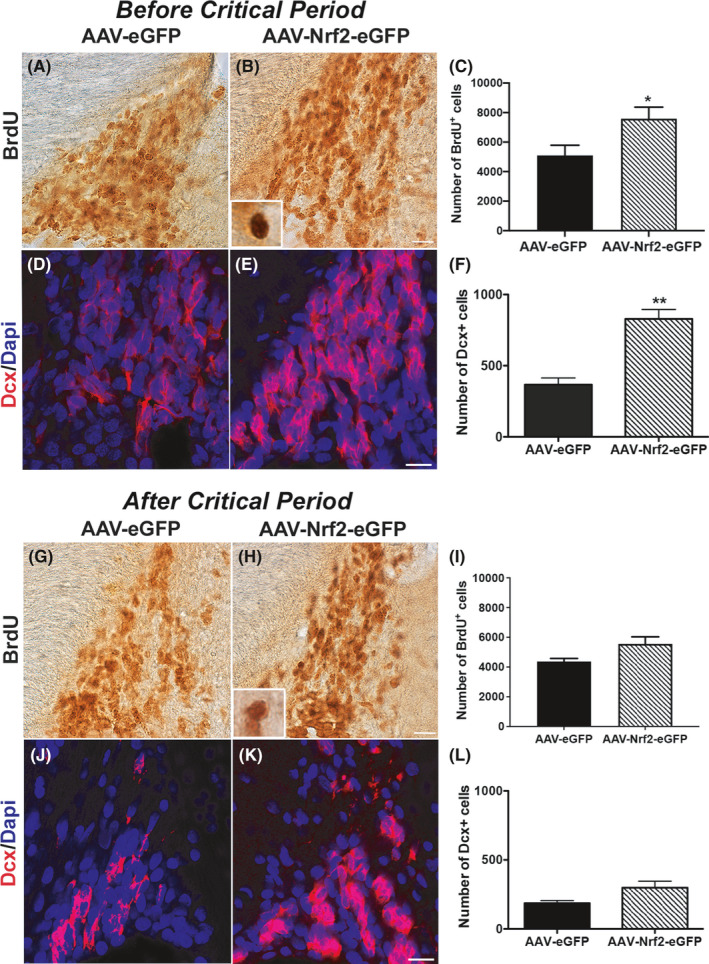
NRF2 upregulation promotes NSPC proliferation and neurogenesis. (A,B) shows a comparison of BrdU staining between 11‐month‐old AAV‐eGFP and AAV‐NRF2‐eGFP‐injected rats (before critical period stage), at 4 mos after viral administration. Comparative Dcx immunostaining is shown in (D,E). Quantification of the number of BrdU^+^ and Dcx^+^ cells is in (C,F). BrdU and Dcx data from older rats which had received AAV‐NRF2‐eGFP at 20 mos of age (again at 4 mos post‐viral delivery) is in (G–I) and (J–L), respectively. [*p < 0.05, **p < 0.01, unpaired *t* tests with Welch's correction]. Insets in (B,H) show higher magnification views of BrdU labeled cells. Scale bars: 20 μM. Scale bar for (A,B) is in (B); for (D,E) is in (E), for (G,H) is in (H); for (J,K) is in (K)

Based on our findings that increased NRF2 expression can promote SVZ‐associated behavioral function, we next investigated the effects of NRF2 transduction on NSPC proliferation and neurogenesis. Bromodeoxyuridine (BrdU; S‐phase marker of dividing NSPCs) and Doublecortin (Dcx, marker of newborn neuroblasts or type A NSPCs) immunostaining was used. It was noted that there were greater numbers of BrdU^+^ cells in the SVZs of rats receiving AAV‐NRF2‐eGFP before the CP, compared to AAV‐eGFP‐injected controls (Figure 3A,B). Unbiased stereological counts using the optical fractionator probe of the StereoInvestigator program confirmed that there was indeed a significant increase in the number of BrdU^+^ cells in the ipsilateral SVZs of AAV‐NRF2‐eGFP‐injected animals than controls (Figure 3C). It was found that there were 7578.3 ± 782.8 BrdU^+^ cells across the rostrocaudal extent of the dorsolateral SVZs of AAV‐NRF2‐eGFP‐injected animals compared to 5097.3 ± 688.4 in AAV‐eGFP controls (*p *< 0.05; *p* = 0.044, unpaired *t* test, *t* = 2.38, df = 8). This correlated to about a 81% increase in BrdU cell numbers in the virus‐injected hemisphere of the AAV‐NRF2‐eGFP animals compared to the non‐injected hemisphere, versus a 2% increase in BrdU cell numbers in the virus‐injected side of AAV‐eGFP controls over their corresponding non‐injected side. In contrast, although a rise in BrdU^+^ cells was also observed in the 20‐month‐old animals receiving AAV‐NRF2‐eGFP compared to controls (Figure 3G–I; 5557.8 ± 479.2 vs. 4380.9 ± 197.7; *p* = 0.052, unpaired *t* test, *t* = 2.27, df = 8), this increase was not statistically significant.

With respect to SVZ neurogenesis, quantification of Dcx^+^ cells along the rostrocaudal axis of the dorsolateral SVZ revealed that 11‐month‐old rats receiving AAV‐NRF2‐eGFP had a higher number of Dcx^+^ cells than their counterpart controls (Figure 3F; *p* = 0.001, unpaired *t* test, *t* = 5.70, df = 6). Confocal fluorescence images of representative Dcx staining are shown in Figure 3D,E. Conversely, 20‐month‐old animals injected with AAV‐NRF2‐eGFP showed a slight but insignificant difference in Dcx^+^ cell numbers (Figure 3J–L). Moreover, BrdU and Dcx cell numbers in HBSS controls were similar to GFP only receiving animals. These data indicate that both NSPC proliferation and neurogenesis were enhanced by NRF2 overexpression during the critical, but not post‐critical, period.

### 2.3 Increased NRF2 supports SVZ NSPC regeneration during the critical period

**FIGURE 4:**
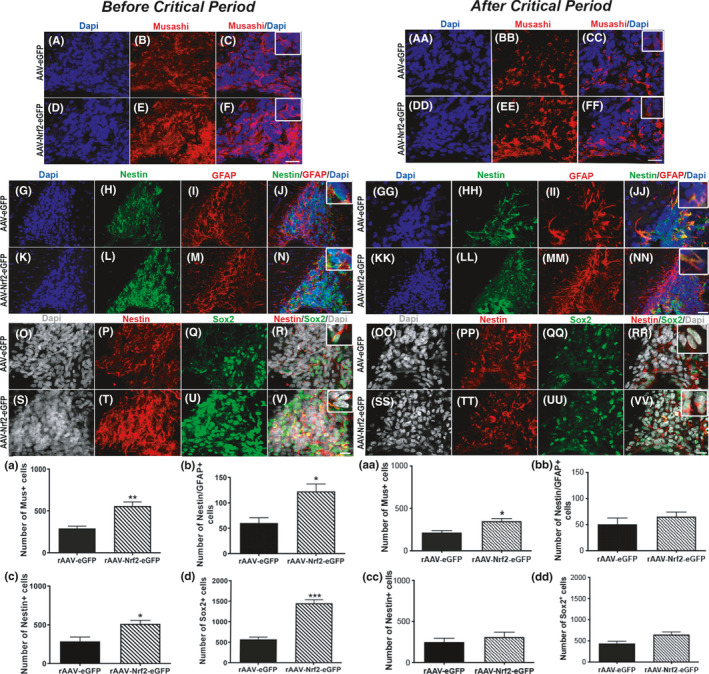
Effects of increased NRF2 expression on the SVZ NSPC regenerative process. The effects of increased NRF2 expression were further studied in rats virally transduced before the advent of the critical period at 11 mos of age or after the critical period (A–F, AA–FF) shows a comparison of Musashi staining in the dorsolateral SVZ 4 mos after viral delivery between AAV‐Nr2‐eGFP and AAV‐eGFP administered rats. High magnification overlay images are in the insets in C, F, CC, FF. Associated quantification is shown in (a) and (aa). Nestin/GFAP and Nestin/Sox2 comparisons are shown in G–N, GG–NN and O–V, OO–VV, respectively, with high magnification insets in N, NN and V, VV. Graphs (b–d**)** and (bb–dd) show a quantification of GFAP/Nestin double‐labeled, Nestin, and Sox2 expressing cells in the two groups. [*p < 0.05, **p < 0.01, ***p < 0.001, unpaired *t* tests with Welch's correction]. Scale bars: 20 μM. Scale bar for A–F is in F; for G–N is in N; for O–V is in V; AA–FF is in FF; for GG–NN is in NN; for OO–VV is in VV

Given that viral NRF2 expression during the critical period increased SVZ NSPC proliferation and neurogenesis, we interrogated how NRF2 affects various NPSC subtypes in the SVZ by examining the expression of markers that delineate different SVZ stem and progenitor cells. SVZ NSPCs show a hierarchy of division: glial‐like type B cells divide relatively infrequently to give rise to rapidly dividing type C transit‐amplifying cells (also referred to as intermediate progenitor cells), which expand the progenitor pool. These type C transit‐amplifying cells then generate immature type A neuroblasts that mature into fully differentiated neurons. To assess these different NSPC subtypes, we first stained for Musashi1 (Mus) that is highly expressed in type B and type C NSPCs. It was observed that Mus immunolabeling (red) in 11‐month‐old AAV‐NRF2‐eGFP‐injected rats was notably greater than in AAV‐eGFP controls (Figure 4A–F). Confocal quantification confirmed higher numbers of Mus^+^ cells in the dorsolateral SVZ of AAV‐NRF2‐eGFP‐injected rats compared to controls (Figure 4A; *p* = 0.003, unpaired *t* test, *t* = 4.72, df = 6). Next, we examined GFAP/Nestin (denotes type B NSPCs), Sox 2 (marker of C and some type B NSPCs), and Nestin (seen in type B and C NSPCs) expression (Figure 4G–V). Consistent with the neurogenesis data in Figure 3, the number of Nestin (green) and GFAP (red) double‐positive NSPCs, as well as Sox2 (green) and Nestin (red) expressing NSPCs, was all significantly increased in the 11‐month‐old AAV‐NRF2‐eGFP‐injected animals as opposed to controls. This increase was further verified by enumerating these specific cell populations via confocal microscopy (Figure 4b–d; GFAP/Nestin – *p* = 0.01, unpaired *t* test, *t* = 3.48, df = 6; Sox2 – *p* = 0.0005, unpaired *t* test, *t* = 8.03, df = 5; Nestin – *p* = 0.02, unpaired *t* test, *t* = 3.21, df = 5). These data, in concert with the data in Figure 3, suggest that NRF2 activation improves the regeneration of all major SVZ cell types, namely type B, type C and type A cells.

In the 20‐month‐old group, AAV‐NRF2‐eGFP‐injected rats showed more prominent Mus immunolabeling in the dorsolateral SVZ, compared to AAV‐NRF2‐eGFP‐injected controls (Figure 4AA–FF). Confocal quantification revealed significantly more Mus^+^ cells in the dorsolateral SVZ of AAV‐NRF2‐eGFP‐injected rats (Figure 4aa; *p* = 0.025, unpaired *t* test, *t* = 3.47, df = 4). However, in contrast to the 11‐month‐old rats, no notable differences were detected in the numbers of GFAP/Nestin (Figure 4GG–NN), Sox 2 and Nestin (Figure 4OO–VV) expressing cells (Figure 4bb–dd). These data indicated that increased NRF2 expression improved NSPC regeneration to some extent in the older animals, but not at all stages of neurogenesis.

### 2.4 Increased NRF2 promotes NSPC migration via the RMS during the critical period

**FIGURE 5:**
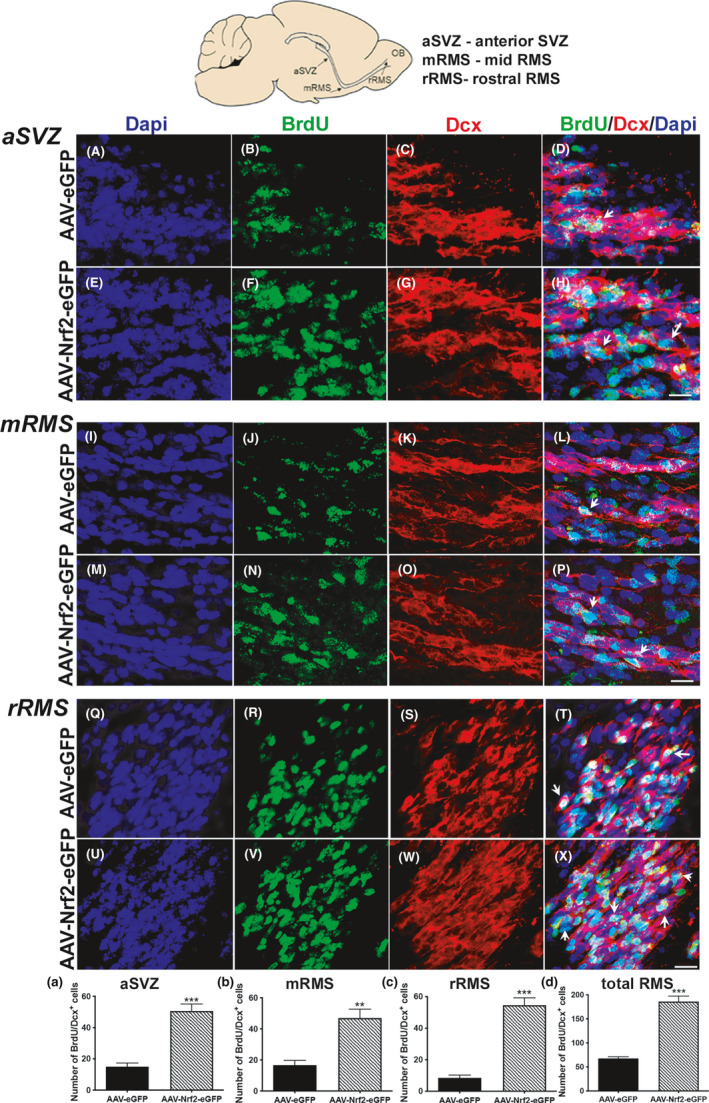
NSPCs travel more successfully through the rostral migratory stream to the olfactory bulb upon NRF2 upregulation. Immunohistochemical analysis of newborn BrdU^+^/Dcx^+^ cells in the aSVZ, mRMS, and rRMS at 4 mos after viral injections in 15‐month‐old AAV‐eGFP and AAV‐Nr2‐eGFP rats was conducted (see schematic at the top). AAV‐NRF2‐eGFP‐injected animals showed higher BrdU/Dcx co‐labeling in the aSVZ (A–H), mRMS (I–P), and rRMS (Q–X), compared to AAV‐eGFP controls (Arrows point to example BrdU/Dcx double‐positive cells). Associated data from confocal quantification are shown in (a–c). (d) Conveys the average number of cells present across all three regions. [*p < 0.05, **p < 0.01, ***p < 0.001, unpaired *t* tests]. Scale bars: 10 μM. Scale bars for A–H is in H; for I–P is in P; for Q–X is in X

Newly generated neuroblasts (type A cells) leave the SVZ from the anterior part (base of the anterior horn of the lateral ventricle) and migrate along the rostral migratory stream (RMS) to the olfactory bulb (OB), where they differentiate into mature olfactory interneurons (Li et al., 2009). More specifically, developing neurons travel caudally and ventrally through the vertical limb of the RMS, after which they turn and follow the horizontal limb, traveling ventrally and rostrally, to reach the anterior olfactory cortex (Martinez‐Molina et al., 2011). To determine what effect viral NRF2 expression had on NSPC migration, we assessed the immature neuroblast movement from the anterior end of the SVZ (aSVZ) where migrating cells start to compact into the RMS, to the mid‐RMS (mRMS) where the stream bends, and finally to the rostral part of the RMS (rRMS) just as it reaches the OB, by tallying newly born BrdU^+^/Dcx^+^ cells present in each region (location of regions is shown in schema in Figure 5). We observed an increased presence of BrdU (green) and Dcx (red) double‐positive cells in the aSVZ (Figure 5A–H), mRMS (Figure 5I–P), and rRMS (Figure 5Q–X) of 11‐month‐old animals injected with AAV‐NRF2‐eGFP compared to control‐injected animals. Cell counts of the total number BrdU^+^/Dcx^+^ cells, in several levels across the rostrocaudal extent of each region (Figure 5a–d) determined that increased NRF2 had promoted greater traversal of newborn cells through the different RMS areas (aSVZ – *p* = 0.0002, unpaired *t* test, *t* = 7.94, df = 6; mRMS – *p* = 0.002, unpaired *t* test, *t* = 4.89, df = 6; rRMS – *p* = 0.001, unpaired *t* test, *t* = 9.82, df = 6). These results suggested that newly generated NSPCs overexpressing NRF2 not only proliferate and regenerate at the level of the SVZ, but also migrate more effectively to the OB, than control cells. When NSPC migration was assessed in the older 20‐month‐old animals in the aSVZ and rRMS, it was found that the number of BrdU^+^/Dcx^+^ expressing cells was higher on average in rats treated with AAV‐NRF2‐eGFP (Figure S2A–R). However, these increases were not statistically significant compared to control rats.

### 2.5 Increased NRF2 expression supports NSPC differentiation and neuronal maturation

**FIGURE 6:**
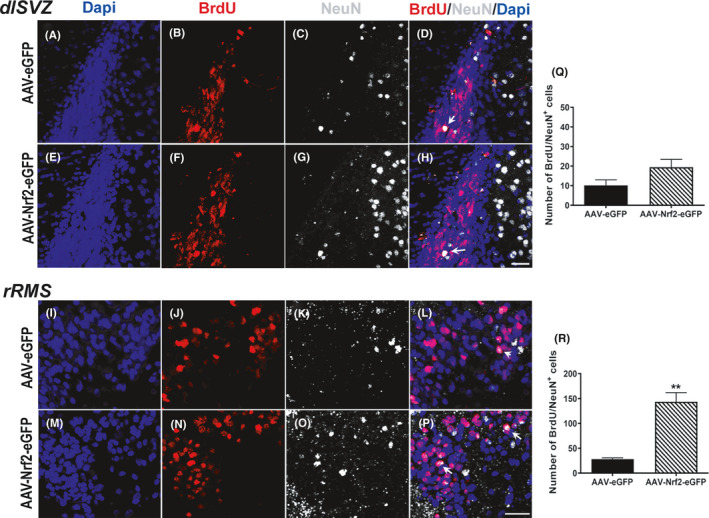
Effects of NRF2 overexpression on neuronal maturation of the NSPCs. Newly born cells which had matured into neurons (BrdU/NeuN co‐expressing cells) were assessed at the level of the dorsolateral SVZ (A‐H), and rostral RMS (I‐P) (Arrows point to example BrdU/NeuN co‐labeled cells). Confocal microscopy‐based quantification of this data is shown in (Q,R). [**p < 0.01, ***p < 0.001, unpaired *t* tests]. Scale bars: 20 μM. Scale bar for (A‐H) is in (H); (I‐P) is in (P)

Having confirmed that an amplification of NRF2 expression improves NSPC proliferation, regeneration, and migration during the critical period, we studied whether these newly generated migratory neurons were capable of maturing as they reached the OB. To address this question, we performed double immunostaining to detect newborn BrdU^+^ cells (red) that have developed into mature neurons (white, NeuN^+^), in both the dorsolateral SVZ and the rostral RMS (Figure 6A–P). Results revealed greater BrdU^+^/NeuN^+^ immunoreactivity in the dorsolateral SVZs of 11‐month‐old animals injected with AAV‐NRF2‐eGFP, compared to controls. However, upon quantification of cell numbers, this increase was not statistically significant (Figure 6Q; *p* = 0.09, unpaired *t* test, *t* = 1.88, df = 8). In contrast, the number of BrdU^+^/NeuN^+^ cells was significantly higher in the rRMS of AAV‐NRF2‐eGFP‐injected rats (Figure 6R; *p* = 0.003, unpaired *t* test, *t* = 6.19, df = 4). On the other hand, no significant increases in the numbers of BrdU^+^/NeuN^+^ cells in the dorsolateral SVZ (Figure S2AA–HH,QQ) or rRMS (Figure S2II–PP,RR) were found in the older 20‐month‐old animals receiving AAV‐NRF2‐eGFP compared to control rats. Altogether, our results indicate that rescuing NRF2 expression during, but not after, the CP improves NSPC proliferation/regeneration, and that these newborn progenitor cells can migrate toward the OB and mature into neurons, to cause a significant improvement in associated behavioral outcomes. A strong upregulation of NRF2 and GCLM in the SVZs of the 20‐month‐old animals (Figure S3A–I) was seen, comparable to that in the younger animals, suggesting that other cell‐intrinsic and/or extrinsic factors maybe interfering with NRF2‐mediated effects in the older animals.

### 2.6 Increased NRF2 expression promotes striatal neurogenesis

**FIGURE 7:**
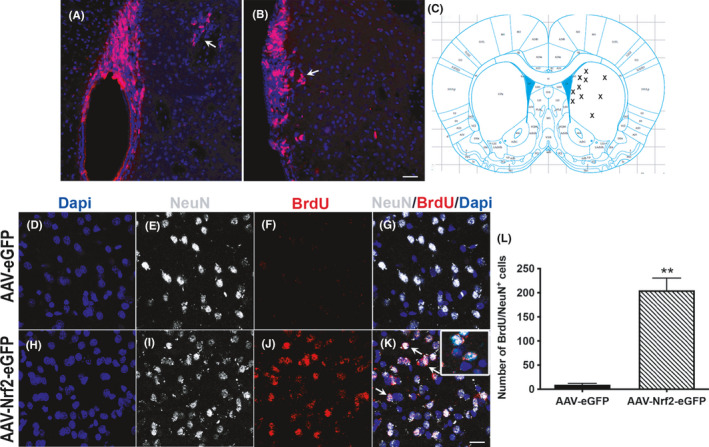
NRF2 overexpression before the CP induces striatal neurogenesis. (A,B) show low magnification views of BrdU labeled cells (arrow in (A) points to groups of Brdu labeled cells in striatum; arrow in (B) points to BrdU cells potentially disengaging from the SVZ). The schematic in (C) depicts the general areas (X marks) where individual BrdU cells were found. (D‐K) show high magnification images of BrdU/NeuN cells in the strata of AAV‐eGFP vs. AAV‐NRF2‐eGFP animals, with quantification in (I). Arrows in (K) point to example BrdU/NeuN co‐expressing cells. [**p < 0.01, unpaired *t* tests]. Scale bar for (A,B) is in (B) (50 μM); (D‐K) is in (K) (15 μM)

Given the significant improvements in motor learning seen at 4 mos after viral NRF2 transduction in the 11‐month‐old rats, we also assessed striatal neurogenesis in the animals. Immunostaining with BrdU showed no apparent streams of potentially migrating cells from the subventricular zone in the AAV‐NRF2‐eGFP‐injected animals, although occasional BrdU cells disjointed from the SVZ as well as pockets of BrdU cells in the striatum were noted (arrows in Figure 7A,B). The BrdU^+^ cells were most often found as single isolated cells distributed predominantly in the dorsomedial, and some in the dorsolateral, striatum (schema in Figure 7C depicts this distribution of BrdU cells). NeuN immunostaining and high‐resolution confocal imaging showed that multiple BrdU cells were co‐expressing the neuronal marker (Figure 7D–K). Quantification determined that there were higher numbers of BrdU^+^/NeuN^+^ double‐stained cells in AAV‐NRF2‐eGFP‐injected animals than in the animals that had received AAV‐eGFP only (Figure 7L). We also examined the differentiation of the newborn neurons into DARPP32^+^ cells, a marker of medium spiny neurons which constitute a large proportion of striatal neurons. However, we did not detect any BrdU labeled cells co‐expressing DARPP32 (Figure S4A–H). These data indicated that increased NRF2 expression before the CP had induced striatal neurogenesis, but not their further subtype specification.

## 3 DISCUSSION

Recent studies have provided information on mechanisms that may drive the aging of NSPCs (Apostolopoulou et al., 2017; Corenblum et al., 2016; Leeman et al., 2018; Moore et al., 2015; Ray et al., 2018). However, less is known on whether NSPC aging can be reversed or invigorated. Our previous work linked the disruption of the stress‐resistance transcription factor, NRF2, to deficits in NSPC function with age (Corenblum et al., 2016; Ray et al., 2018). The current study investigated whether rescuing NRF2 expression could counter age‐related NSPC deficits. We find that, indeed, amplifying NRF2 expression can positively regulate NSPC function during aging. In particular, our results demonstrate that NRF2 activation, especially before a certain critical period of vulnerability during aging, can enhance NSPC regeneration and function. These novel data are the first to reveal the ability of a cell‐intrinsic factor, namely NRF2, to control NSPC aging and impact lifelong neural plasticity.

Three major results arise from the current work. Firstly, at a cellular level, the study determines that NRF2 upregulation can significantly promote the activity of aging NSPCs. The data show that viral expression of NRF2 at 11 mos of age alleviates the decline in SVZ NSPC regeneration normally seen during the 13–15 mos critical period. Specifically, this increase in NRF2 expression stimulated (1) NSPC proliferation (BrdU^+^ cells), (2) self‐renewal (GFAP^+^/Nestin^+^ type B cells), and (3) progressive differentiation through the production of type C transit‐amplifying cells (Nestin^+^, Sox2^+^ cells) and subsequently type A neuroblasts (Dcx^+^ cells). Moreover, the migration of newborn Dcx^+^ neuroblasts through the RMS, and their neuronal maturation (into NeuN^+^ cells) was also enhanced. These data indicate that revitalizing NRF2 expression during the critical aging period can improve NSPC regeneration at all major stages of SVZ neurogenesis.

Secondly, our data show that the observed activation of NSPC regeneration, upon NRF2 upregulation, correlated with a significantly better performance on fine olfactory discrimination and motor learning tasks—thus connecting molecular enhancements to a behaviorally relevant readout. It was noted that 11‐month‐old rats that had received control AAV viruses displayed an expected decline in olfactory discrimination function by 15 mos of age. However, rats that were administered AAVs encoding NRF2 exhibited superior olfactory discrimination abilities at 2 mos and 4 mos post‐viral delivery (at 13 and 15 mos aging stages). This suggests that NRF2 upregulation can not only induce increased NSPC proliferation, self‐renewal, differentiation, and migration to the OB, but also affect the olfactory circuitry leading to functional effects. In terms of motor function, AAV‐NRF2‐eGFP‐injected rats showed higher composite motor scores and faster traverse times on a beam walking task. Specifically, NRF2 overexpressing animals were able to cross the narrower 20 and 15 mm beams without foot slips or falls, compared to control animals. The AAV‐NRF2‐eGFP animals also traveled across the 15 mm beam at a quicker pace than controls. These data suggest that NRF2‐based activation of NSPCs can also support striatum‐based motor function. In this regard, it is known that a recruitment of newborn cells into the striatum (which is adjacent to the SVZ) can occur under conditions of increased NSPC regeneration and neurogenesis (Benraiss et al., 2012; Kobayashi et al., 2006; Madhavan et al., 2012; Yamashita et al., 2006). Our data show that this was indeed the case in the NRF2 overexpressing rats, thus providing a basis for the improvement in motor learning in the 11‐month‐old animals.

A third important finding is that the supportive effects of NRF2 on NSPC activity and function were largely muted when viral NRF2 delivery was delayed until an older age of 20 mos (after the end of the CP). More specifically, although Musashi expressing cells were increased, and an almost significant increase in BrdU cell numbers (*p *= 0.052) was observed upon NRF2 upregulation in the older rats, other major NSPC populations (Nestin/GFAP, Nestin/Sox2, Dcx, and their behavior) were largely not affected. These results identify a certain age‐ and time‐dependency of NRF2 effects, which is intriguing. More broadly, our data suggest that specific downstream molecular events may already have taken root, to irrevocably compromise NSPC function, by the end of the critical middle‐aged period, thus contributing to a resistance to NRF2‐based rejuvenation after this time. Dysfunction of proteasome‐dependent proteolysis is also heavily implicated in aging and cell senescence (Leeman et al., 2018; Morimoto & Cuervo, 2009). NRF2 regulates proteasome expression, and its activation has been shown to impede cellular senescence, while inactivation of the pathway recapitulates aging phenotypes (Gabriel et al., 2015; Kubben et al., 2016; Schmidlin et al., 2019). Such mechanisms may be important in determining the age‐dependent NRF2 effects seen in our study. Interestingly, other studies have indicated that old mouse NSPCs can be activated through specific intrinsic manipulations (Leeman et al., 2018; Seib et al., 2013). In the context of the current work, since NRF2 has a multitude of targets besides GCLM, it is possible that age‐associated differences exist in the ability of NRF2 to activate certain downstream genes versus others due to cell‐intrinsic or other extrinsic influences coming from the older niche. For instance, age‐related epigenetic alterations in the NRF2 pathway may be involved (Guo et al., 2015). Such processes will need to be further investigated. Nevertheless, our work provides important information regarding specific time‐periods during which NSPCs may be more amenable, or resistant, to change—a fundamental subject that needs to be understood but one on which not much is known.

In the larger context of the presented work, we comment that although NRF2 is known as major transcription factor, essential to the cell's survival and homeostatic mechanisms, its specific contribution and importance in stem cells is only recently emerging (Bryan et al., 2013; Schmidlin et al., 2019; Suzuki & Yamamoto, 2017). Stem cells, including pluripotent/embryonic stem cells and adult tissue stem cells, possess unique metabolic programs and reduction–oxidation (or redox) states to sustain proliferation while maintaining pluripotency, multipotency, and/or specified differentiation (Dai et al., 2020). In this vein, it has been shown that NRF2 may govern stem cell function through the modulation of redox and metabolic pathways involving mitochondria and the proteasome (Holmstrom et al., 2016; Jang et al., 2014). In particular, NRF2, by its ability to control cellular reactive oxygen species (ROS) levels, would promote an optimal intracellular redox environment increasingly recognized as critical to stem cell function (Hochmuth et al., 2011; Madhavan, 2015; Noble et al., 2003; Rafalski & Brunet, 2011). Studies by Khacho et al. (2016) suggest that changes in mitochondrial dynamics during neural stem cell development regulate cell fate decisions through a ROS‐dependent NRF2‐mediated transcriptional process (Khacho et al., 2016). The metabolic reprogramming from oxidative phosphorylation to glycolytic energy production seen during the induction of pluripotent stem cell differentiation is also dependent on ROS‐mediated NRF2 activation (Hawkins et al., 2016; Zhou et al., 2016). Besides these metabolic and redox effects, NRF2 is also known to directly regulate cell division and phenotypic fate by interacting with other transcription factors and cell cycle regulators involved in maintaining cellular self‐renewal, multipotency/pluripotency, and differentiation (Wakabayashi et al., 2015; Zhu et al., 2013). Our work aligns with these studies and highlights NRF2 as a crucial player in aging stem cells.

In conclusion, our study provides evidence that enriching NRF2 expression during a critical time of aging can meaningfully support NSPC activity and function. These data implicate NRF2 as a powerful age‐relevant regulator of NSPCs. Understanding the molecular basis of NRF2’s effects will reveal fundamental aspects of NSPC biology that underlie its ability to sustain enduring plasticity and lifelong resilience. Moreover, optimizing NRF2 pathway regulation of downstream targets will likely expand the opportunities for clinical translation of NSPCs.

## 4 EXPERIMENTAL PROCEDURES

### 4.1 Animals

Adult male Fisher 344 rats aged 11 mos and 20 mos were obtained from the National Institutes of Health (NIH‐NIA). The rats were housed in the University Animal Care Facility at The University of Arizona, under a reverse 12‐h light‐dark cycle condition with food and water available ad libitum. Animals were treated according to the rules and regulations of the NIH and Institutional Guidelines on the Care and Use of Animals. The University of Arizona Institutional Animal Care and Use Committee (IACUC) approved all experimental procedures.

### 4.2 Experimental design, viral injections, and BrdU administration

SVZ NSPCs were transduced with recombinant adeno‐associated viral vectors serotype 2/1 (AAV2/1) encoding NRF2 (pAAV‐CMV‐Nfe2l2‐IRES‐eGFP) or eGFP (pAAV‐CMV‐eGFP) as a control. As described before, the AAV viruses were generated at the Children's Hospital of Philadelphia Viral Vector Core (https://ccmt.research.chop.edu/cores_rvc.php) (Ray et al., 2018). Viral transduction occurred in 11‐month‐old and 20‐month‐old rats, in which the vectors were administered unilaterally into the SVZ via stereotaxic methods, at a dose of 1.18 × 10^12^ vg/ml. The vectors were injected into two sites (1.5 μls) along the rostrocaudal axis of the SVZ (Coordinates: AP +1.2, ML +1.2, DV −3.6 and AP +0, ML +2, DV −4; Figure S1). Animals injected with only HBSS were also included as controls.

The number of animals in each experimental group was as follows:

11‐month‐old animals: AAV‐eGFP (n = 9); AAV‐NRF2‐eGFP (n = 9); HBSS (n = 6). 20‐month‐old animals: AAV‐eGFP (n = 9); AAV‐NRF2‐eGFP (n = 9); HBSS (n = 6). The overall experimental design is shown in Figure 1A.

Animals were sacrificed four mos after AAV administration, at 15 and 24 mos of age. All animals were sacrificed via pentobarbital (60 mg/kg) and perfusion with 4% paraformaldehyde (PFA; Electron Microscopy Sciences). Subsequently, brains were extracted and post‐fixed in 4% PFA solution, sunk through a 30% sucrose solution, and sectioned in the coronal (40 μm) or sagittal (36 μm) plane on a freezing sliding microtome for morphological studies (Corenblum et al., 2016; Madhavan et al., 2015; Ray et al., 2018).

Intraperitoneal Bromodeoxyuridine (BrdU) injections were given at about 4 mos post‐AAV to label proliferating and migrating NSPCs (Corenblum et al., 2015; Madhavan et al., 2015). BrdU was delivered at a dose of 50 mg/kg/12 h for 3 days, and the animals sacrificed 4 days afterward.

#### 4.2.1 Behavioral analysis

##### Fine olfactory discrimination behavior

Rats were subjected to behavioral testing via a fine olfactory discrimination task, which is an established measure of the SVZ NSPC function and neurogenesis in vivo (Corenblum et al., 2016; Enwere et al., 2004). As described previously, the task includes an initial training and subsequent testing stages for Discrete and Fine Olfactory Discrimination abilities after water restriction for 48 h (a 1 h period of access to water/day was allowed). Briefly, for the training stage, 12 μl of double distilled water was placed in a sterile dish, and 1 μl of coconut extract (COC) was added. This combination served as a reward for response and was designated [+]. The dish was placed at one end of the cage, and the rat was allowed 2 min to find and drink the [+]. Once the rat finished drinking, the dish was removed and replaced with a fresh [+] solution after a 30‐s inter‐trial interval. The amount of COC was increased with each trial until it reached 8.5 μl per dish per trial. From here, five trials were conducted using [+]. For the sixth trial, we presented the rats with culture dishes containing 8.5 μl of almond extract (ALM) applied to the surface of 12 μl of a 1% solution of denatonium benzoate (DB: Sigma‐Aldrich). This combination of ALM and DB was designated [−]. Because DB is extremely bitter, the rats found it aversive and learned to associate the bitter taste with the smell of ALM. They subsequently avoided drinking the [−]. Four additional trials were conducted with [−] to ensure that the rats had learned to avoid the [−]. In the fine discrimination testing, the COC and ALM in the [+] and [−], respectively, were replaced with various mixtures of both COC and ALM to produce a graph of performance against odor concentration known as a generalization gradient. Rats were required to discriminate between solutions containing [+] ratios of COC:ALM of 100:0 (basic discrete discrimination), 60:40, 58:42, and 56:44 as well as the corresponding [−] ratios of COC:ALM of 0:100, 40:60, 42:58 and 44:56. The first trial utilizing the [+] mixture and its corresponding [−] mixture was conducted with the same 2‐min time limit and 30‐s inter‐trial interval. This was repeated so that five total trials were conducted.

##### Challenging beam test

In order to assess striatum‐based motor function, rats were tested through a modified beam walking task (Drucker‐Colin & Garcia‐Hernandez, 1991). Briefly, rats were trained to traverse a set of wooden beams of three different widths (15 mm, 20 mm, and 30 mm) consisting of a start platform at 100 cm above floor height, and an end platform (with rat's home cage) also at 100 cm above floor height. The rats were then evaluated through different measures to analyze motor strength and coordination. Training and testing are performed in the dark during animal's awake cycle. Briefly, training consists of 3 days, where each animal is placed on the beam and allowed to traverse the length in each of three trials. A 30‐s rest time is allowed between runs. Testing consisted of the rat being placed on each of the three beams consecutively and allowed to traverse the length. Two trials were completed for each width of beam. If the animal did not cross the beam in 120 s, the trial was considered unsuccessful. Evaluations are based on successful beam crossings, total time to traverse the beam, and foot slip errors. Specifically, quantification of total traverse time was taken, in seconds, and consisted of only trials where an animal successfully crossed the entire beam and did not fall. For an overall task assessment, a composite score was also generated giving each animal a starting score of 3. One point each was subtracted if the animal made a foot slip error or scooted across the beam instead of using its limbs to cross. If the animal fell or did not cross the beam, it was given a score of zero.

### 4.3 Immunohistochemistry

Sections were blocked [10% normal goat serum, 0.5% triton‐X‐100 in tris‐buffered saline (TBS, pH 7.4)] and incubated in primary antibody overnight at room temperature (RT). Primary antibodies were detected in a 2‐h incubation at RT with secondary antibodies coupled to fluorochromes Alexa 488, 594, 647 (Life Technologies‐Molecular Probes) and counterstained with 4′,6′‐diamidino‐2‐phenylindole, dihydrochloride (DAPI, Life Technologies). Alternatively, a chromogenic method was used in which primaries were exposed to biotinylated secondary antibodies (Vector Laboratories) followed by treatment with ABC reagent (Vector Laboratories) and 3′‐Diaminobenzidine (DAB: Sigma‐Aldrich). Control conditions constituted the deletion of the primary antibody or secondary antibody and the inclusion of relevant isotype specific antibodies and sera instead of the omitted antibodies. Primary antibodies used were as follows: NRF2 (1:100, Santa Cruz Biotechnology); GFP (1:200, Abcam); GCLM (1:200, Santa Cruz Biotechnology); Nestin (1:10, Developmental Studies Hybridoma Bank); Sox2 (1:500, Abcam); BrdU (1:100, Abcam); Musashi (1:300, MilliporeSigma); NeuN (1:500, MilliporeSigma); Dcx (1:500, Abcam); GFAP (1:500, MilliporeSigma); Darpp32 (1:3000, Cell Signaling). [A more detailed description of the antibodies used is provided in Table S1].

#### 4.3.1 Stereology and cell counting

##### BrdU stereology

Stereological probes were applied using a Zeiss Imager M2 microscope (Carl Zeiss) equipped with StereoInvestigator software (v2019.1.3; MBF Bioscience) according to previously published methods (Madhavan et al., 2012, 2015). Using the optical fractionator probe, BrdU cell counts were conducted through the dorsolateral SVZ in sections at 480 μm intervals across the rostrocaudal axis of the structure. In terms of the dorsoventral extent of the SVZ counted, it covered the SVZ area to a point midway between the genu of the corpus callosum and the anterior commissure crossing. In all cases, after section thickness was determined, guard zones were set at 2 μm each at the top and bottom of the section. All contours were drawn around the region of interest at 2.5x magnification. Clear uniformly labeled nuclei were counted under a 63x oil immersion objective using a grid size of 40 × 40 μm and a counting frame size of 60 × 60 μm. The counting frame was lowered at 1–2 μm interludes and each cell in focus was marked. The Gundersen method for calculating the coefficient of error was used to estimate the accuracy of the optical fractionator results. Coefficients obtained were generally less than 0.10.

##### Other cell counts

The number of SVZ NSPCs expressing Musashi, Nestin/GFAP, Nestin/Sox2, and Dcx was enumerated in confocal z‐stacks (1 µm intervals) taken through the dorsolateral SVZ across its rostrocaudal extent (40 μm thick coronal sections, 1:12 series, five sections per animal, n = 5 animals/group). Positive cells were counted using Zen Blue (v2.5) software (Zeiss). The number of BrdU/Dcx and BrdU/NeuN cells along the rostrocaudal extent RMS was counted in 36 μm sagittal sections (1:12 series, five sections per animal, n = 5 animals/group) sagittal sections (1:6 series, three sections/animal, n = 3 animals/group). Z‐stacks (1 µm intervals) were taken in three specific regions—anterior border of SVZ, mid‐RMS, and rostral RMS (aSVZ, mRMS, and rRMS, respectively; see Figure 5). Striatal BrdU/NeuN cell counts were conducted in 40 μm thick coronal sections (1:12 series, five sections per animal, n = 3 animals/group). NRF2/GCLM counts were conducted in in 40 μm thick coronal sections (three adjacent sections per animal, n = 3 animals/group). Cells were counted using markers in Zen Blue. Data were expressed as mean ± SEM of the total number of cells obtained across rostral to caudal sections counted in each experimental group.

### 4.4 Microscopy

Fluorescence analysis was performed using a Zeiss LSM880 confocal microscope (Zeiss). Z sectioning was performed at 1–2 μm intervals in order to verify the co‐localization of markers. Image extraction and analysis was conducted via the Zen Blue software (v2.5; Zeiss). A Zeiss M2 Imager microscope connected to an AxioCam Mrc digital camera connected was used for brightfield microscopy. A Leica DMI 6000 inverted microscope (Leica Microsystems) equipped with Leica Application Suite‐Advanced Fluorescence 3.0 and a Hamamatsu Flash 4.0 sCMOS greyscale camera was used to image entire sections using a 5X dry objective. These pictures were captured using the Leica LAS‐X version 3.7 software (Leica Microsystems) and used to generate stitched images that showed broader views of the AAV‐eGFP transduction.

### 4.5 Statistical analyses

Sigmaplot 11 and GraphPad prism 8 software were used for statistical analyses. For comparing two groups, *t* tests were used. For comparisons between three or more groups, one‐way analysis of variance (ANOVA) followed by Tukey's or Bonferroni's post hoc test for multiple comparisons between treatment groups was conducted. A two‐way repeated measures ANOVA with Tukey's post hoc test was applied to the olfactory behavioral data. Differences were accepted as significant at *p* < 0.05. Statistical details as pertaining to each experiment are provided within the relevant results and legend sections.

## CONFLICT OF INTEREST

The authors declare no competing financial interests.

## AUTHOR CONTRIBUTIONS

AA – Conception and design, Collection and assembly of data, Data analysis and Interpretation, Manuscript writing. KK – Collection and assembly of data, Data analysis, Manuscript editing. MJC – Collection and assembly of data, Data analysis, Manuscript editing. LM – Conception and design, Collection and assembly of data, Data analysis and Interpretation, Manuscript writing, Financial support, Final approval of manuscript.

## Supporting information

Fig S1

Fig S2

Fig S3

Fig S4

Table S1

## Data Availability

The data that support the findings of this study are available from the corresponding author upon reasonable request. As such, we will follow guidance provided by the journal for sharing the data.

## References

[acel13385-bib-0001] Alvarez‐Buylla, A. , & Lim, D. A. (2004). For the long run: Maintaining germinal niches in the adult brain. Neuron, 41(5), 683–686. 15003168 10.1016/s0896-6273(04)00111-4

[acel13385-bib-0002] Apostolopoulou, M. , Kiehl, T. R. , Winter, M. , Cardenas De La Hoz, E. , Boles, N. C. , Bjornsson, C. S. , Zuloaga, K. L. , Goderie, S. K. , Wang, Y. , Cohen, A. R. , & Temple, S. (2017). Non‐monotonic changes in progenitor cell behavior and gene expression during aging of the adult V‐SVZ neural stem cell niche. Stem Cell Reports, 9(6), 1931–1947. 10.1016/j.stemcr.2017.10.005 29129683 PMC5785674

[acel13385-bib-0003] Benraiss, A. , Bruel‐Jungerman, E. , Lu, G. , Economides, A. N. , Davidson, B. , & Goldman, S. A. (2012). Sustained induction of neuronal addition to the adult rat neostriatum by AAV4‐delivered noggin and BDNF. Gene Therapy, 19(5), 483–493. 10.1038/gt.2011.114 21918547 PMC3655807

[acel13385-bib-0004] Bond, A. M. , Ming, G. L. , & Song, H. (2015). Adult mammalian neural stem cells and neurogenesis: Five decades later. Cell Stem Cell, 17(4), 385–395. 10.1016/j.stem.2015.09.003 26431181 PMC4683085

[acel13385-bib-0005] Bryan, H. K. , Olayanju, A. , Goldring, C. E. , & Park, B. K. (2013). The Nrf2 cell defence pathway: Keap1‐dependent and ‐independent mechanisms of regulation. Biochemical Pharmacology, 85(6), 705–717. 10.1016/j.bcp.2012.11.016 23219527

[acel13385-bib-0006] Conover, J. C. , & Shook, B. A. (2011). Aging of the subventricular zone neural stem cell niche. Aging and Disease, 2(1), 49–63. 22396866 PMC3295044

[acel13385-bib-0007] Corenblum, M. J. , Flores, A. J. , Badowski, M. , Harris, D. T. , & Madhavan, L. (2015). Systemic human CD34(+) cells populate the brain and activate host mechanisms to counteract nigrostriatal degeneration. Regenerative Medicine, 10(5), 563–577. 10.2217/rme.15.32 26237701

[acel13385-bib-0008] Corenblum, M. J. , Ray, S. , Remley, Q. W. , Long, M. , Harder, B. , Zhang, D. D. , Barnes, C. A. , & Madhavan, L. (2016). Reduced Nrf2 expression mediates the decline in neural stem cell function during a critical middle‐age period. Aging Cell, 15(4), 725–736. 10.1111/acel.12482 27095375 PMC4933666

[acel13385-bib-0009] Dai, X. , Yan, X. , Wintergerst, K. A. , Cai, L. , Keller, B. B. , & Tan, Y. (2020). Nrf2: Redox and metabolic regulator of stem cell state and function. Trends in Molecular Medicine, 26(2), 185–200. 10.1016/j.molmed.2019.09.007 31679988

[acel13385-bib-0010] Drucker‐Colin, R. , & Garcia‐Hernandez, F. (1991). A new motor test sensitive to aging and dopaminergic function. Journal of Neuroscience Methods, 39(2), 153–161. 10.1016/0165-0270(91)90081-a 1798345

[acel13385-bib-0011] Encinas, J. M. , Michurina, T. V. , Peunova, N. , Park, J.‐H. , Tordo, J. , Peterson, D. A. , Fishell, G. , Koulakov, A. , & Enikolopov, G. (2011). Division‐coupled astrocytic differentiation and age‐related depletion of neural stem cells in the adult hippocampus. Cell Stem Cell, 8(5), 566–579. 10.1016/j.stem.2011.03.010 21549330 PMC3286186

[acel13385-bib-0012] Enwere, E. , Shingo, T. , Gregg, C. , Fujikawa, H. , Ohta, S. , & Weiss, S. (2004). Aging results in reduced epidermal growth factor receptor signaling, diminished olfactory neurogenesis, and deficits in fine olfactory discrimination. Journal of Neuroscience, 24(38), 8354–8365. 10.1523/JNEUROSCI.2751-04.2004 15385618 PMC6729689

[acel13385-bib-0013] Gabriel, D. , Roedl, D. , Gordon, L. B. , & Djabali, K. (2015). Sulforaphane enhances progerin clearance in Hutchinson‐Gilford progeria fibroblasts. Aging Cell, 14(1), 78–91. 10.1111/acel.12300 25510262 PMC4326906

[acel13385-bib-0014] Guo, Y. , Yu, S. , Zhang, C. , & Kong, A. N. (2015). Epigenetic regulation of Keap1‐Nrf2 signaling. Free Radical Biology and Medicine, 88(Pt B), 337–349. 10.1016/j.freeradbiomed.2015.06.013 26117320 PMC4955581

[acel13385-bib-0015] Hawkins, K. E. , Joy, S. , Delhove, J. M. K. M. , Kotiadis, V. N. , Fernandez, E. , Fitzpatrick, L. M. , Whiteford, J. R. , King, P. J. , Bolanos, J. P. , Duchen, M. R. , Waddington, S. N. , & McKay, T. R. (2016). NRF2 orchestrates the metabolic shift during induced pluripotent stem cell reprogramming. Cell Reports, 14(8), 1883–1891. 10.1016/j.celrep.2016.02.003 26904936 PMC4785773

[acel13385-bib-0016] Hochmuth, C. E. , Biteau, B. , Bohmann, D. , & Jasper, H. (2011). Redox regulation by Keap1 and Nrf2 controls intestinal stem cell proliferation in Drosophila. Cell Stem Cell, 8(2), 188–199. 10.1016/j.stem.2010.12.006 21295275 PMC3035938

[acel13385-bib-0017] Holmström, K. M. , Baird, L. , Zhang, Y. , Hargreaves, I. , Chalasani, A. , Land, J. M. , Stanyer, L. , Yamamoto, M. , Dinkova‐Kostova, A. T. , & Abramov, A. Y. (2013). Nrf2 impacts cellular bioenergetics by controlling substrate availability for mitochondrial respiration. Biology Open, 2(8), 761–770. 10.1242/bio.20134853 23951401 PMC3744067

[acel13385-bib-0018] Holmstrom, K. M. , Kostov, R. V. , & Dinkova‐Kostova, A. T. (2016). The multifaceted role of Nrf2 in mitochondrial function. Current Opinion in Toxicology, 1, 80–91. 10.1016/j.cotox.2016.10.002 28066829 PMC5193490

[acel13385-bib-0019] Itoh, K. , Mimura, J. , & Yamamoto, M. (2010). Discovery of the negative regulator of Nrf2, Keap1: A historical overview. Antioxidants & Redox Signaling, 13(11), 1665–1678. 10.1089/ars.2010.3222 20446768

[acel13385-bib-0020] Jang, J. , Wang, Y. , Kim, H. S. , Lalli, M. A. , & Kosik, K. S. (2014). Nrf2, a regulator of the proteasome, controls self‐renewal and pluripotency in human embryonic stem cells. Stem Cells, 32(10), 2616–2625. 10.1002/stem.1764 24895273 PMC4165656

[acel13385-bib-0021] Katsimpardi, L. , & Lledo, P. M. (2018). Regulation of neurogenesis in the adult and aging brain. Current Opinion in Neurobiology, 53, 131–138. 10.1016/j.conb.2018.07.006 30077888

[acel13385-bib-0022] Khacho, M. , Clark, A. , Svoboda, D. S. , Azzi, J. , MacLaurin, J. G. , Meghaizel, C. , Sesaki, H. , Lagace, D. C. , Germain, M. , Harper, M.‐E. , Park, D. S. , & Slack, R. S. (2016). Mitochondrial dynamics impacts stem cell identity and fate decisions by regulating a nuclear transcriptional program. Cell Stem Cell, 19(2), 232–247. 10.1016/j.stem.2016.04.015 27237737

[acel13385-bib-0023] Kobayashi, T. , Ahlenius, H. , Thored, P. , Kobayashi, R. , Kokaia, Z. , & Lindvall, O. (2006). Intracerebral infusion of glial cell line‐derived neurotrophic factor promotes striatal neurogenesis after stroke in adult rats. Stroke, 37(9), 2361–2367. 16873711 10.1161/01.STR.0000236025.44089.e1

[acel13385-bib-0024] Kubben, N. , Zhang, W. , Wang, L. , Voss, T. C. , Yang, J. , Qu, J. , Liu, G.‐H. , & Misteli, T. (2016). Repression of the antioxidant NRF2 pathway in premature aging. Cell, 165(6), 1361–1374. 10.1016/j.cell.2016.05.017 27259148 PMC4893198

[acel13385-bib-0025] Leeman, D. S. , Hebestreit, K. , Ruetz, T. , Webb, A. E. , McKay, A. , Pollina, E. A. , Dulken, B. W. , Zhao, X. , Yeo, R. W. , Ho, T. T. , Mahmoudi, S. , Devarajan, K. , Passegué, E. , Rando, T. A. , Frydman, J. , & Brunet, A. (2018). Lysosome activation clears aggregates and enhances quiescent neural stem cell activation during aging. Science, 359(6381), 1277–1283. 10.1126/science.aag3048 29590078 PMC5915358

[acel13385-bib-0026] Li, X. , Tang, X. , Jablonska, B. , Aguirre, A. , Gallo, V. , & Luskin, M. B. (2009). p27(KIP1) regulates neurogenesis in the rostral migratory stream and olfactory bulb of the postnatal mouse. Journal of Neuroscience, 29(9), 2902–2914. 10.1523/JNEUROSCI.4051-08.2009 19261886 PMC3488282

[acel13385-bib-0027] Liu, L. , & Rando, T. A. (2011). Manifestations and mechanisms of stem cell aging. Journal of Cell Biology, 193(2), 257–266. 10.1083/jcb.201010131 PMC3080271 21502357

[acel13385-bib-0028] Madhavan, L. (2015). Redox‐based regulation of neural stem cell function and Nrf2. Biochemical Society Transactions, 43(4), 627–631. 10.1042/BST20150016 26551703

[acel13385-bib-0029] Madhavan, L. , Daley, B. F. , Davidson, B. L. , Boudreau, R. L. , Lipton, J. W. , Cole‐Strauss, A. , Steece‐Collier, K. , & Collier, T. J. (2015). Sonic hedgehog controls the phenotypic fate and therapeutic efficacy of grafted neural precursor cells in a model of nigrostriatal neurodegeneration. PLoS One, 10(9), e0137136. 10.1371/journal.pone.0137136 26340267 PMC4560385

[acel13385-bib-0030] Madhavan, L. , Daley, B. F. , Sortwell, C. E. , & Collier, T. J. (2012). Endogenous neural precursors influence grafted neural stem cells and contribute to neuroprotection in the parkinsonian rat. European Journal of Neuroscience, 35(6), 883–895. 10.1111/j.1460-9568.2012.08019.x PMC3719977 22417168

[acel13385-bib-0031] Malhotra, D. , Portales‐Casamar, E. , Singh, A. , Srivastava, S. , Arenillas, D. , Happel, C. , Shyr, C. , Wakabayashi, N. , Kensler, T. W. , Wasserman, W. W. , & Biswal, S. (2010). Global mapping of binding sites for Nrf2 identifies novel targets in cell survival response through ChIP‐Seq profiling and network analysis. Nucleic Acids Research, 38(17), 5718–5734. 10.1093/nar/gkq212 20460467 PMC2943601

[acel13385-bib-0032] Martinez‐Molina, N. , Kim, Y. , Hockberger, P. , & Szele, F. G. (2011). Rostral migratory stream neuroblasts turn and change directions in stereotypic patterns. Cell Adhesion and Migration, 5(1), 83–95. 10.4161/cam.5.1.13788 21045564 PMC3038103

[acel13385-bib-0033] Mirescu, C. , & Gould, E. (2006). Stress and adult neurogenesis. Hippocampus, 16(3), 233–238. 10.1002/hipo.20155 16411244

[acel13385-bib-0034] Moore, D. L. , Pilz, G. A. , Arauzo‐Bravo, M. J. , Barral, Y. , & Jessberger, S. (2015). A mechanism for the segregation of age in mammalian neural stem cells. Science, 349(6254), 1334–1338. 10.1126/science.aac9868 26383951

[acel13385-bib-0035] Morimoto, R. I. , & Cuervo, A. M. (2009). Protein homeostasis and aging: Taking care of proteins from the cradle to the grave. Journals of Gerontology. Series A, Biological Sciences and Medical Sciences, 64(2), 167–170. 10.1093/gerona/gln071 PMC2655025 19228787

[acel13385-bib-0036] Nilsson, M. , Perfilieva, E. , Johansson, U. , Orwar, O. , & Eriksson, P. S. (1999). Enriched environment increases neurogenesis in the adult rat dentate gyrus and improves spatial memory. Journal of Neurobiology, 39(4), 569–578. 10.1002/(sici)1097-4695(19990615)39:4<569:aid-neu10>3.0.co;2-f 10380078

[acel13385-bib-0037] Noble, M. , Smith, J. , Power, J. , & Mayer‐Proschel, M. (2003). Redox state as a central modulator of precursor cell function. Annals of the New York Academy of Sciences, 991, 251–271. 12846992 10.1111/j.1749-6632.2003.tb07481.x

[acel13385-bib-0038] Rafalski, V. A. , & Brunet, A. (2011). Energy metabolism in adult neural stem cell fate. Progress in Neurobiology, 93(2), 182–203. 10.1016/j.pneurobio.2010.10.007 21056618

[acel13385-bib-0039] Ray, S. , Corenblum, M. J. , Anandhan, A. , Reed, A. , Ortiz, F. O. , Zhang, D. D. , Barnes, C. A. , & Madhavan, L. (2018). A role for Nrf2 expression in defining the aging of hippocampal neural stem cells. Cell Transplantation, 27(4), 589–606. 10.1177/0963689718774030 29871525 PMC6041888

[acel13385-bib-0040] Schmidlin, C. J. , Dodson, M. B. , Madhavan, L. , & Zhang, D. D. (2019). Redox regulation by NRF2 in aging and disease. Free Radical Biology and Medicine, 134, 702–707. 10.1016/j.freeradbiomed.2019.01.016 30654017 PMC6588470

[acel13385-bib-0041] Seib, D. R. M. , Corsini, N. S. , Ellwanger, K. , Plaas, C. , Mateos, A. , Pitzer, C. , Niehrs, C. , Celikel, T. , & Martin‐Villalba, A. (2013). Loss of Dickkopf‐1 restores neurogenesis in old age and counteracts cognitive decline. Cell Stem Cell, 12(2), 204–214. 10.1016/j.stem.2012.11.010 23395445

[acel13385-bib-0042] Suzuki, T. , & Yamamoto, M. (2017). Stress‐sensing mechanisms and the physiological roles of the Keap1‐Nrf2 system during cellular stress. Journal of Biological Chemistry, 292(41), 16817–16824. 10.1074/jbc.R117.800169 PMC5641889 28842501

[acel13385-bib-0043] Sykiotis, G. P. , & Bohmann, D. (2008). Keap1/Nrf2 signaling regulates oxidative stress tolerance and lifespan in Drosophila. Developmental Cell, 14(1), 76–85. 10.1016/j.devcel.2007.12.002 18194654 PMC2257869

[acel13385-bib-0044] Tullet, J. M. , Hertweck, M. , An, J. H. , Baker, J. , Hwang, J. Y. , Liu, S. , Oliveira, R. P. , Baumeister, R. , & Blackwell, T. K. (2008). Direct inhibition of the longevity‐promoting factor SKN‐1 by insulin‐like signaling in *C. elegans* . Cell, 132(6), 1025–1038. 10.1016/j.cell.2008.01.030 18358814 PMC2367249

[acel13385-bib-0045] van Praag, H. , Christie, B. R. , Sejnowski, T. J. , & Gage, F. H. (1999). Running enhances neurogenesis, learning, and long‐term potentiation in mice. Proceedings of the National Academy of Sciences USA, 96(23), 13427–13431. 10.1073/pnas.96.23.13427 PMC23964 10557337

[acel13385-bib-0046] Wakabayashi, N. , Chartoumpekis, D. V. , & Kensler, T. W. (2015). Crosstalk between Nrf2 and Notch signaling. Free Radical Biology and Medicine, 88, 158–167. 10.1016/j.freeradbiomed.2015.05.017 26003520 PMC4628857

[acel13385-bib-0047] Wakabayashi, N. , Shin, S. , Slocum, S. L. , Agoston, E. S. , Wakabayashi, J. , Kwak, M.‐K. , Misra, V. , Biswal, S. , Yamamoto, M. , & Kensler, T. W. (2010). Regulation of notch1 signaling by nrf2: Implications for tissue regeneration. Science Signalling, 3(130), ra52. 10.1126/scisignal.2000762 PMC2932745 20628156

[acel13385-bib-0048] Wiesner, D. , Merdian, I. , Lewerenz, J. , Ludolph, A. C. , Dupuis, L. , & Witting, A. (2013). Fumaric acid esters stimulate astrocytic VEGF expression through HIF‐1alpha and Nrf2. PLoS One, 8(10), e76670. 10.1371/journal.pone.0076670 24098549 PMC3789659

[acel13385-bib-0049] Yamashita, T. , Ninomiya, M. , Hernandez Acosta, P. , Garcia‐Verdugo, J. M. , Sunabori, T. , Sakaguchi, M. , Adachi, K. , Kojima, T. , Hirota, Y. , Kawase, T. , Araki, N. , Abe, K. , Okano, H. , & Sawamoto, K. (2006). Subventricular zone‐derived neuroblasts migrate and differentiate into mature neurons in the post‐stroke adult striatum. Journal of Neuroscience, 26(24), 6627–6636. 10.1523/JNEUROSCI.0149-06.2006 16775151 PMC6674034

[acel13385-bib-0050] Zhou, G. , Meng, S. , Li, Y. , Ghebre, Y. T. , & Cooke, J. P. (2016). Optimal ROS signaling is critical for nuclear reprogramming. Cell Reports, 15(5), 919–925. 10.1016/j.celrep.2016.03.084 27117405 PMC4856580

[acel13385-bib-0051] Zhu, J. , Wang, H. , Sun, Q. , Ji, X. , Zhu, L. , Cong, Z. , Zhou, Y. , Liu, H. , & Zhou, M. (2013). Nrf2 is required to maintain the self‐renewal of glioma stem cells. BMC Cancer, 13, 380. 10.1186/1471-2407-13-380 23937621 PMC3751732

