# Supplementary figures and images for: Enhanced NRF2 expression mitigates the decline in neural stem cell function during aging
Source: Aging Cell. 2021 Jun 15;20(6):e13385. doi: 10.1111/acel.13385 (PMC8208782; doi:10.1111/acel.13385)

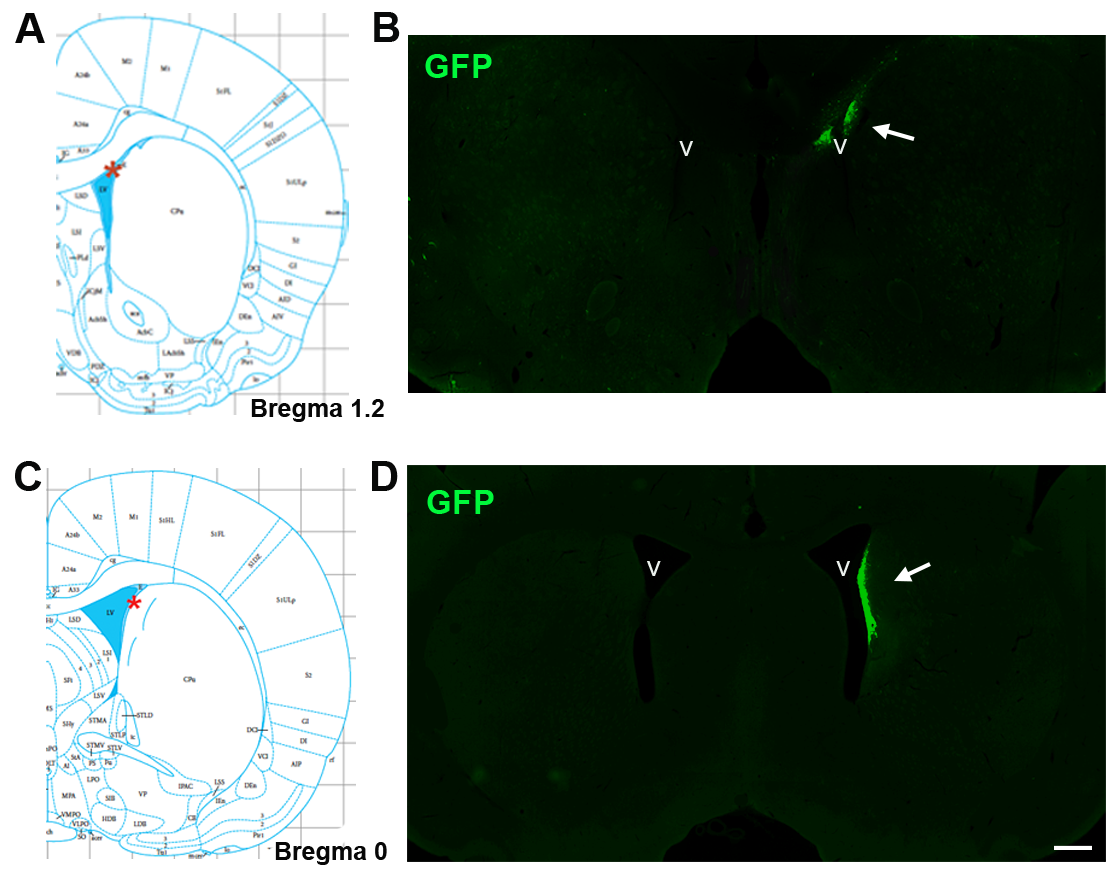

Supplement: Supplementary file 1 — Fig S1 [file ACEL-20-e13385-s001.tif]

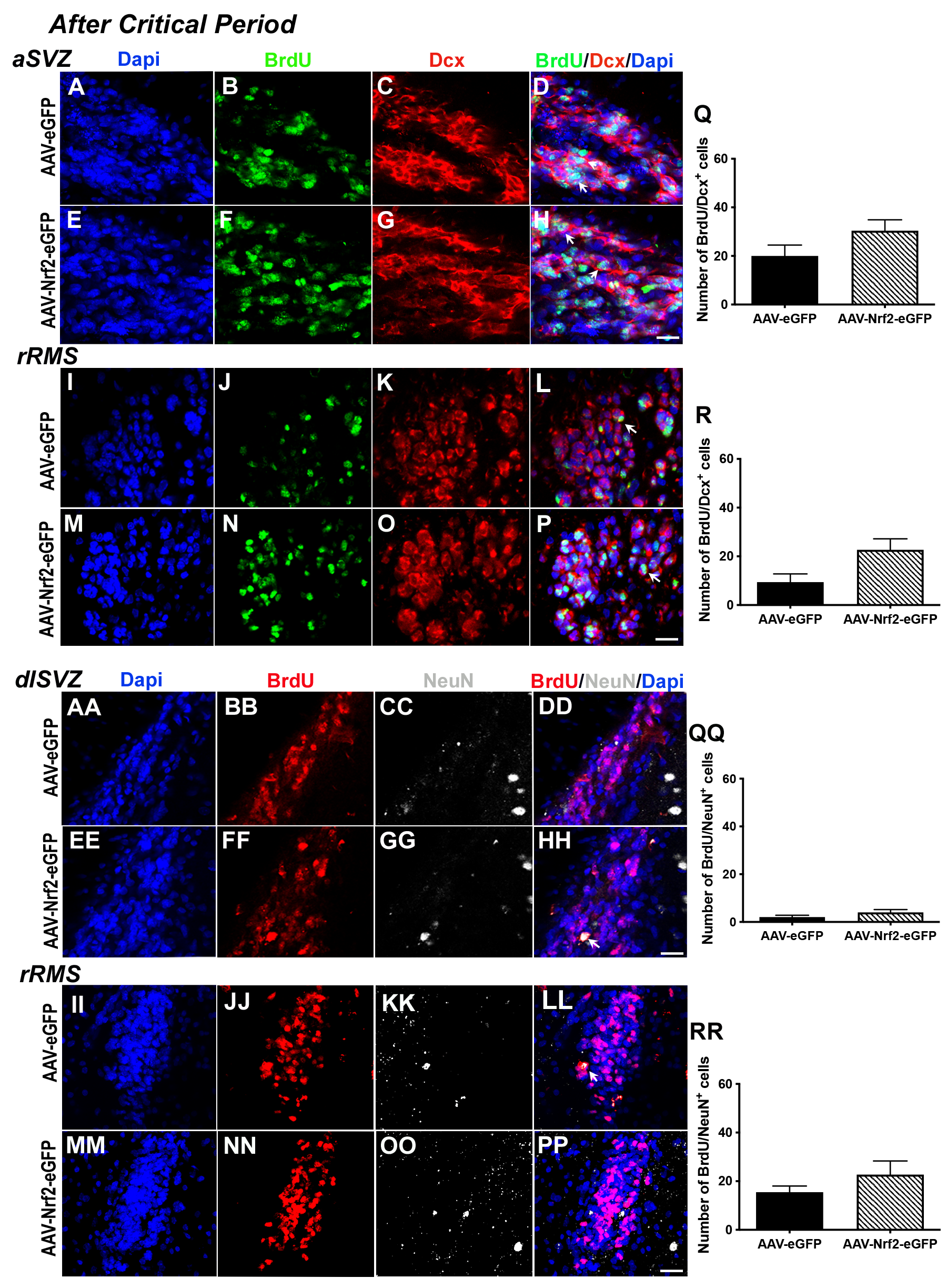

Supplement: Supplementary file 2 — Fig S2 [file ACEL-20-e13385-s002.tif]

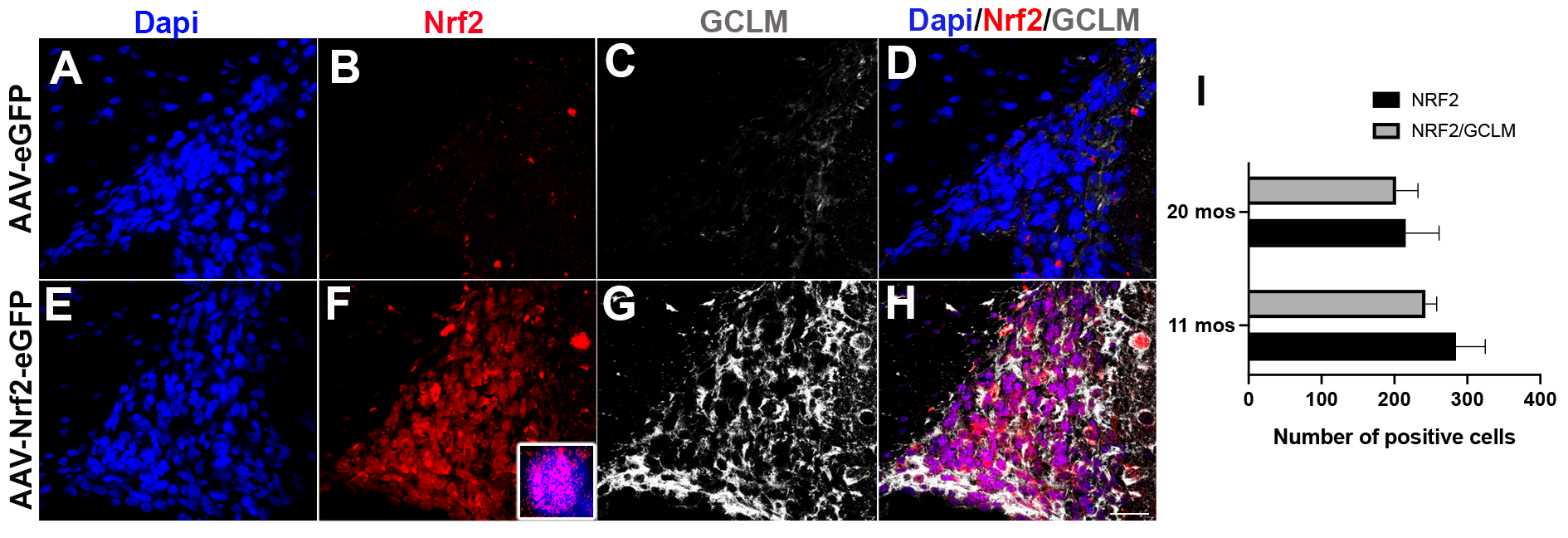

Supplement: Supplementary file 3 — Fig S3 [file ACEL-20-e13385-s004.tif]

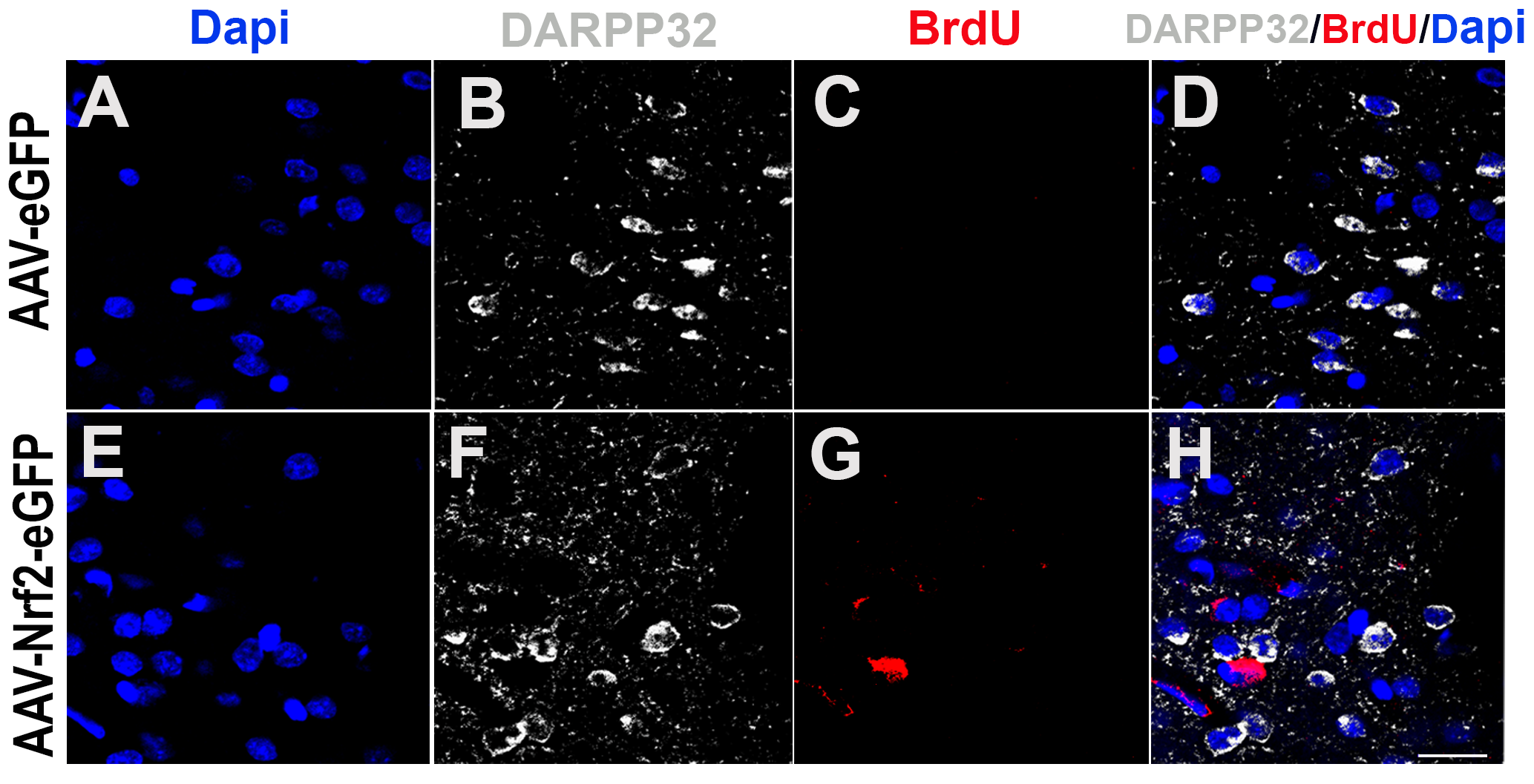

Supplement: Supplementary file 4 — Fig S4 [file ACEL-20-e13385-s003.tif]
