# Supplementary material for: Enhanced NRF2 expression mitigates the decline in neural stem cell function during aging
Source: Aging Cell. 2021 Jun 15;20(6):e13385. doi: 10.1111/acel.13385 (PMC8208782; doi:10.1111/acel.13385)
Supplement: Supplementary file 5 — Table S1 [file ACEL-20-e13385-s005.docx]

**Supplementary Table 1:**

| anti-Sox2 | Abcam | Ab97959 | Rabbit/Polyclonal |
| --- | --- | --- | --- |
| anti-cGCSm (E-4) | Santa Cruz | sc-55586 | Mouse/Monoclonal |
| anti-Glial Fibrillary Acidic Protein (GFAP), Clone: GA5 | Millipore | MAB360 | Mouse/Monoclonal |
| anti-Musashi-1 | Millipore | AB5977 | Rabbit/Polyclonal |
| anti-NRF2, H-300 | Santa Cruz | SC-13032 | Rabbit/Polyclonal |
| anti-Bromodeoxyuridine, IgG2a BU1/75 (ICR1) | Abcam | ab6326 | Rat/Monoclonal |
| anti-Nestin, Clone: rat-401 | DHSB | Rat-401 | Mouse/Monoclonal |
| anti-Neuronal Nuclei (NeuN), Clone: A60 | Millipore | MAB377 | Mouse/Monoclonal |
| anti-Doublecortin (Dcx) | Abcam | Ab2253 | Guinea Pig/Polyclonal |
| Anti-DARPP32 (19A3) | Cell Signaling | 2306 | Rabbit/Monoclonal |
